# Supplementary material for: Youth-associated protein TIMP2 alters microglial state and function in the context of aging
Source: bioRxiv. 2025 Jun 8:2025.05.20.655226. Originally published 2025 May 21. Preprint. [Version 2] doi: 10.1101/2025.05.20.655226 (PMC12139801; doi:10.1101/2025.05.20.655226)
Supplement: Supplement 1 [file NIHPP2025.05.20.655226v2-supplement-1.pdf]

bioRxiv preprint doi: https://doi.org/10.1101/2025.05.20.655226; this version posted June 9, 2025. The copyright holder for this preprint (which was not certified by peer review) is the author/funder, who has granted bioRxiv a license to display the preprint in perpetuity. It is made available under aCC-BY 4.0 International license.

Supplementary Figure 1

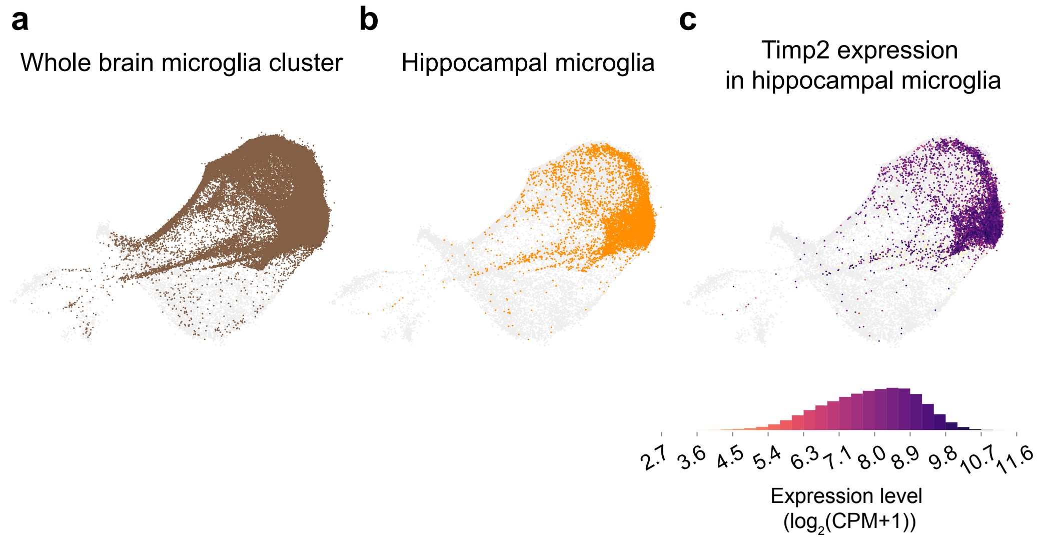

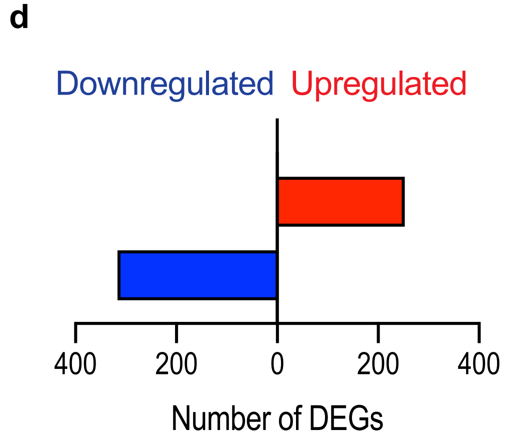

e

Canonical pathways	Predicted activation	*P*-value
Synaptogenesis signaling pathway	Inhibited	1.21E-04
Apoptosis signaling	Inhibited	9.16E-04
Synaptic long-term potentiation	Inhibited	2.49E-03
Rac signaling	Inhibited	4.59E-03
Senescence pathway	Activated	1.89E-03
Interferon gamma signaling	Activated	2.49E-03

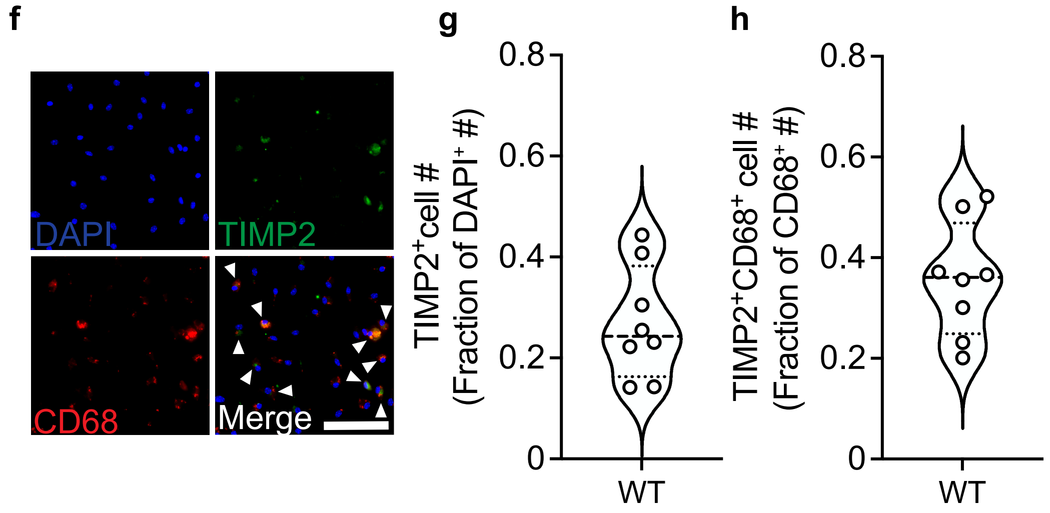

bioRxiv preprint doi: https://doi.org/10.1101/2025.05.20.655226; this version posted June 9, 2025. The copyright holder for this preprint (which was not certified by peer review) is the author/funder, who has granted bioRxiv a license to display the preprint in perpetuity. It is made available under aCC-BY 4.0 International license.

Supplementary Figure 1. Microglia express TIMP2 protein near lysosomes.

(a) Data from Allen Brain Cell scRNAseq and MERFISH Atlas representing whole brain microglia (brown), with subsets corresponding to **(b)** hippocampal microglia (orange), and **(c)** TIMP2 expression within hippocampal microglia. **(d)** Number of upregulated and downregulated DEGs in TIMP2 KO microglia relative to WT microglia (N = 7 samples per genotype, with each sample representing a pool of 5 pups (P6-P7); sex-matched). **(e)** IPA canonical pathways based on upregulated and downregulated DEGs in TIMP2 KO and WT microglia from panel (d), with corresponding predicted inhibition or activation with *P*-values. **(f)** Representative images from WT primary microglia cultures stained by ICC with DAPI, as well as anti-TIMP2 and anti-CD68 antibodies for overlap analyses (N = 8 mice, 5-6 month-old mice; sex-matched; 2 independent experiments combined; scale bar = 100 µm), with **(g)** quantification of the number of TIMP2$^+$ cells as a fraction of DAPI$^+$ cells, or **(h)** the number of TIMP2$^+$CD68$^+$ cells as a fraction of cells expressing CD68. Violin plots of median with quartiles.

bioRxiv preprint doi: https://doi.org/10.1101/2025.05.20.655226; this version posted June 9, 2025. The copyright holder for this preprint (which was not certified by peer review) is the author/funder, who has granted bioRxiv a license to display the preprint in perpetuity. It is made available under aCC-BY 4.0 International license.

1122 Supplementary Figure 2

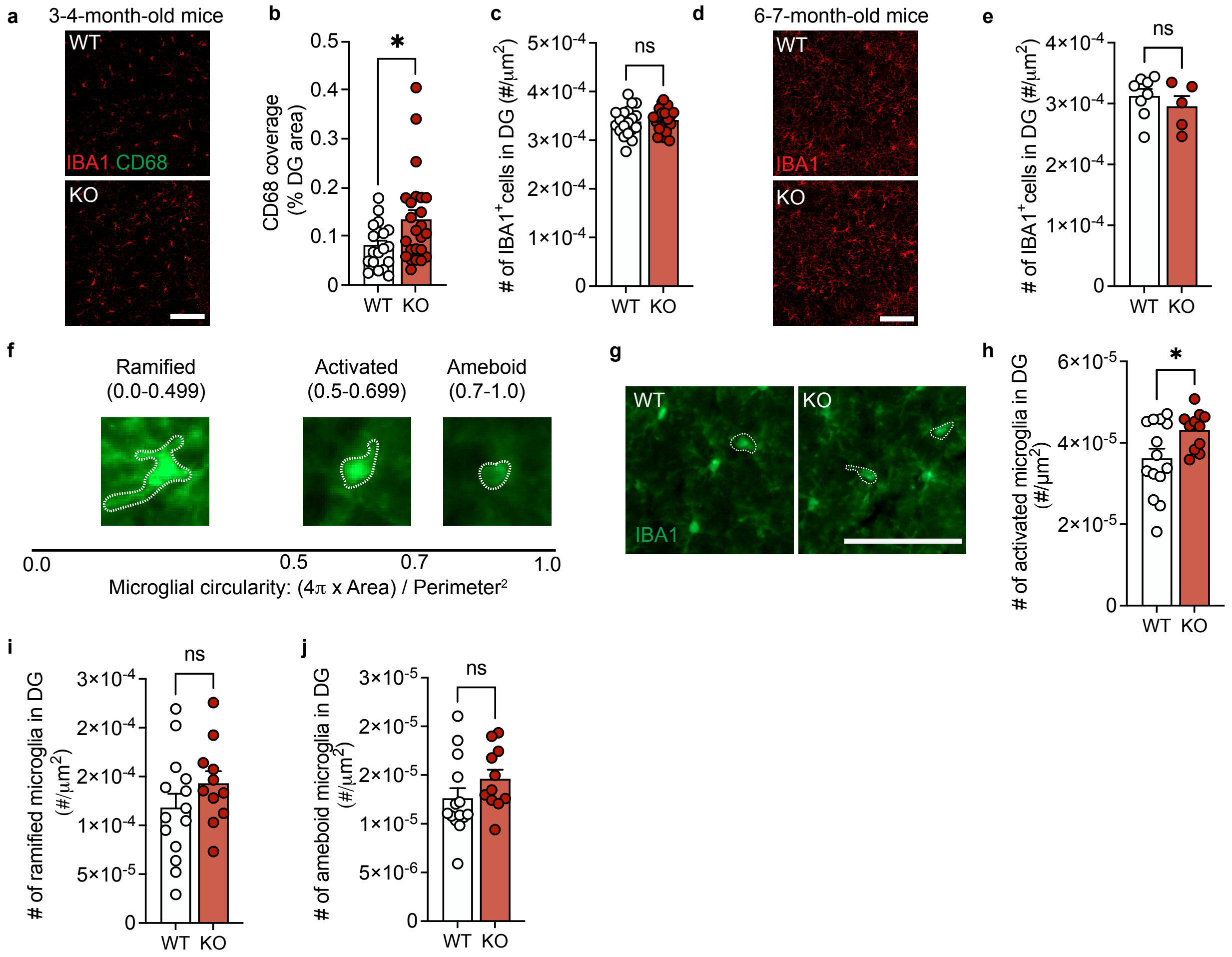

bioRxiv preprint doi: https://doi.org/10.1101/2025.05.20.655226; this version posted June 9, 2025. The copyright holder for this preprint (which was not certified by peer review) is the author/funder, who has granted bioRxiv a license to display the preprint in perpetuity. It is made available under aCC-BY 4.0 International license.

Supplementary Figure 2. TIMP2 KO mice exhibit altered state and morphology associated with activation.

(a) Representative confocal images of DG from 3-4-month-old WT and TIMP2 KO mice stained with anti-CD68 and anti-IBA1 antibodies (scale bar = 100 μm) with **(b)** quantification of thresholded DG area covered by $CD68^+$ staining and **(c)** the number of $IBA1^+$ cells normalized to DG area (N = 9-13 mice per group; sex-matched; mean ± SEM). **(d)** Representative confocal images of DG from 6-7-month-old WT and TIMP2 KO mice stained with anti-IBA1 antibody (scale bar = 100 μm) with **(e)** quantification of the number of $IBA1^+$ cells normalized to DG area (N = 5-8 mice per group; female; mean ± SEM). **(f)** Representation of morphology analysis based on microglia circularity, with representative images for each morphological state according to circularity scale. **(g)** Representative microscopy images of DG from 6-7-month-old WT and TIMP2 KO mice stained with anti-IBA1 antibodies, with white dashed lines outlining microglia with "activated" morphology and corresponding (scale bar = 100 μm) **(h)** quantification of number of microglia with "activated" microglia (circularity = 0.5-0.699) per DG area. **(i)** Quantification of the number of microglia with "ramified" morphology (circularity = 0.0-0.499) per DG area, and **(j)** the number of microglia with "ameboid" microglia (circularity = 0.7-1.0) per DG area. (N = 11-14 mice per group; sex-matched; mean ± SEM). $^*P < 0.05$; Student's t test; n.s., not significant.

bioRxiv preprint doi: https://doi.org/10.1101/2025.05.20.655226; this version posted June 9, 2025. The copyright holder for this preprint (which was not certified by peer review) is the author/funder, who has granted bioRxiv a license to display the preprint in perpetuity. It is made available under aCC-BY 4.0 International license.

1140 Supplementary Figure 3

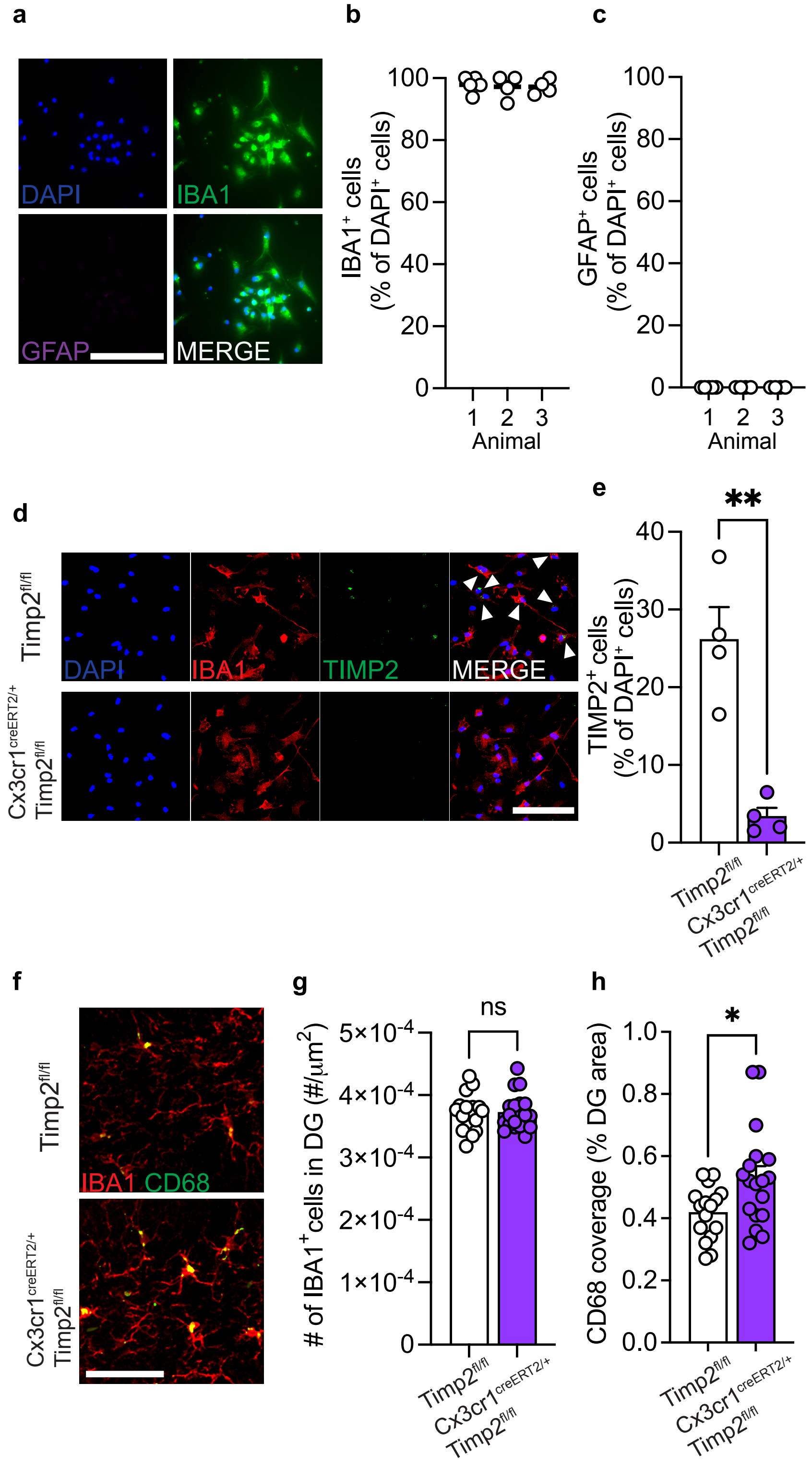

bioRxiv preprint doi: https://doi.org/10.1101/2025.05.20.655226; this version posted June 9, 2025. The copyright holder for this preprint (which was not certified by peer review) is the author/funder, who has granted bioRxiv a license to display the preprint in perpetuity. It is made available under aCC-BY 4.0 International license.

Supplementary Figure 3. Purity of primary microglia cultures and efficiency of microglia *Timp2* deletion

(a) Representative microscopy images of primary microglia cultures from WT mice stained with DAPI, anti-IBA1, and anti-GFAP antibodies (scale bar = 100 μm) with **(b)** quantification of IBA1$^+$ cells as a percentage of DAPI$^+$ cells or **(c)** quantification of GFAP$^+$ cells as a percentage of DAPI$^+$ cells (N = 4-5 replicates per mouse from N = 3 mice). **(d)** Representative confocal images from primary microglia cultures from 5-month-old Timp2$^{fl/fl}$ control and Cx3cr1$^{CreERT2/+}$;Timp2$^{fl/fl}$ littermates stained with DAPI, anti-IBA1 antibody, and anti-TIMP2 antibody (arrowheads indicate TIMP2$^+$ microglia; N= 4 mice per group; sex-matched; scale bar = 100 μm) with **(e)** quantification of TIMP2$^+$ cells as a percentage of DAPI$^+$ cell number (mean ± SEM). **(f)** Representative confocal images of DG from 6-7-month-old TIMP2$^{fl/fl}$ control and Cx3cr1$^{CreERT2/+}$;Timp2$^{fl/fl}$ littermates stained with anti-IBA1 and anti-CD68 antibodies (N= 17-19 mice per group; sex-matched; scale bar = 50 μm) with **(g)** quantification of the number of IBA1$^+$ cells per DG area and **(h)** quantification of DG area covered by CD68$^+$ staining (mean ± SEM). *P < 0.05; **P < 0.01; n.s., not significant. Student's t test.

bioRxiv preprint doi: https://doi.org/10.1101/2025.05.20.655226; this version posted June 9, 2025. The copyright holder for this preprint (which was not certified by peer review) is the author/funder, who has granted bioRxiv a license to display the preprint in perpetuity. It is made available under aCC-BY 4.0 International license.

1156 Supplementary Figure 4

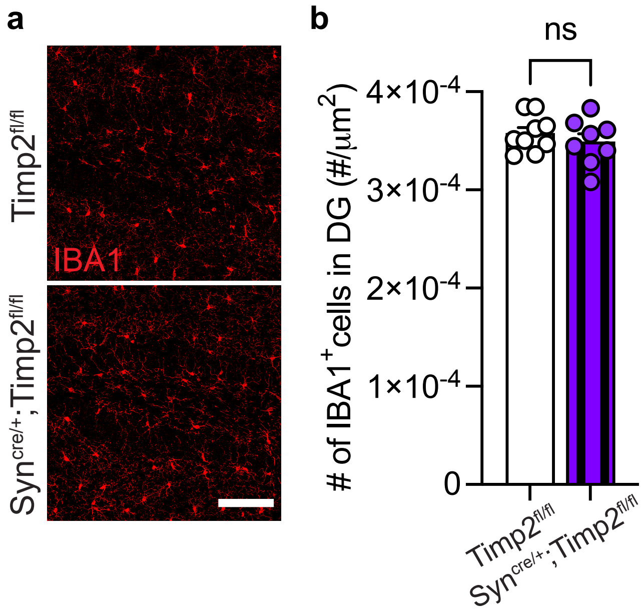

bioRxiv preprint doi: https://doi.org/10.1101/2025.05.20.655226; this version posted June 9, 2025. The copyright holder for this preprint (which was not certified by peer review) is the author/funder, who has granted bioRxiv a license to display the preprint in perpetuity. It is made available under aCC-BY 4.0 International license.

Supplementary Figure 4. Neuronal deletion of TIMP2 does not alter number of microglia in the dentate gyrus

(a) Representative confocal images of DG from *Timp2*$^{fl/fl}$ and Syn$^{Cre/+}$;*Timp2*$^{fl/fl}$ littermates stained with anti-IBA1 antibody with corresponding **(b)** quantification of the number of IBA1^{+} cells normalized to DG area (N = 8-9 mice per group, 2-3-month-old female mice; scale bar = 100 μm). mean ± SEM. ; n.s., not significant. Student's t test.

bioRxiv preprint doi: https://doi.org/10.1101/2025.05.20.655226; this version posted June 9, 2025. The copyright holder for this preprint (which was not certified by peer review) is the author/funder, who has granted bioRxiv a license to display the preprint in perpetuity. It is made available under aCC-BY 4.0 International license.

Supplementary figure 5

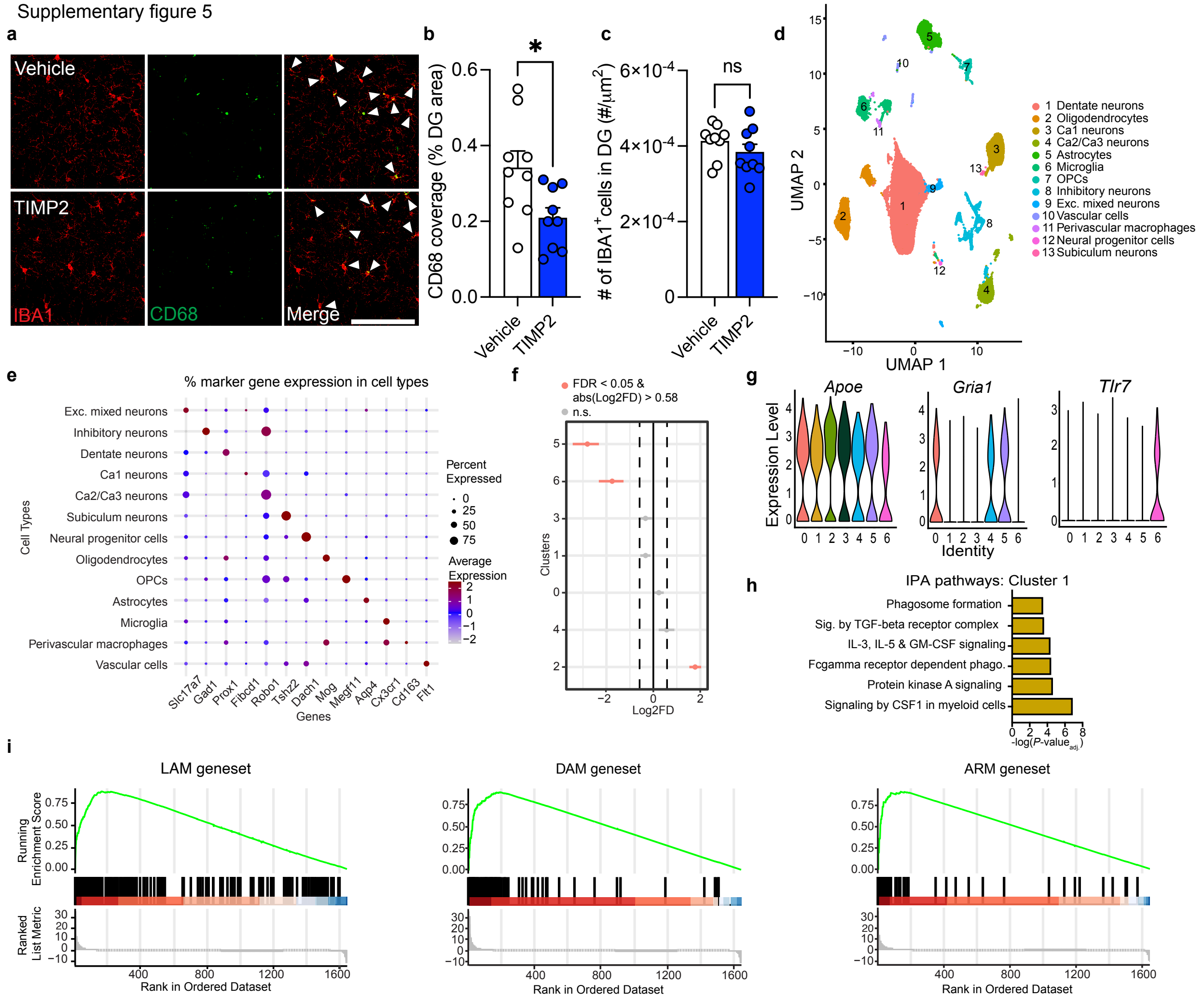

bioRxiv preprint doi: https://doi.org/10.1101/2025.05.20.655226; this version posted June 9, 2025. The copyright holder for this preprint (which was not certified by peer review) is the author/funder, who has granted bioRxiv a license to display the preprint in perpetuity. It is made available under aCC-BY 4.0 International license.

Supplementary Figure 5. Systemic TIMP2 treatment in aged mice alters microglial state and shifts microglial subclusters.

(a) Representative confocal images of DG from 20-month-old WT mice treated systemically with TIMP2 or vehicle, stained with anti-IBA1 and anti-CD68 antibodies (scale bar = 100 μm; white arrowheads indicate $IBA1^+CD68^+$ cells), with corresponding **(b)** quantification of the percentage of DG area covered by $CD68^+$ staining and **(c)** quantification of the number of $IBA1^+$ cells normalized to DG area (N = 9 male mice per group; mean ± SEM). **(d)** Uniform Manifold Approximation and Projection (UMAP) plot of hippocampal nuclei isolated from 20-month-old WT mice treated systemically with vehicle or TIMP2 (N = 4 male mice per group combined as 2 samples per group). **(e)** Dot plot of scaled expression of selected marker genes across annotated cell types. Dot size represents percentage of cells expressing the gene within each cell type, while color indicates the scaled average expression level of the gene across cells in the respective cluster. **(f)** Differences in cell proportions for each cluster between treatment conditions. Significant clusters are denoted in salmon (FDR < 0.05. Log_2 fold-enrichment > 0.58, relative to vehicle-treated mice). **(g)** Violin plots of selected marker genes across microglial subclusters from Fig. 5h. **(h)** Significant canonical pathways from IPA for marker genes significantly upregulated in subcluster 1. **(i)** GSEA enrichment plots for LAM, DAM, and ARM gene sets based on the ranked list of microglial genes (TIMP2 vs. vehicle). Green lines represent running enrichment score, with vertical black bars indicating the position of genes from indicated gene sets in the ranked list and gene ranking direction indicated by color bar (red = upregulated, blue = downregulated).
